# *Anacyclus pyrethrum* (L): Chemical Composition, Analgesic, Anti-Inflammatory, and Wound Healing Properties

**DOI:** 10.3390/molecules25225469

**Published:** 2020-11-23

**Authors:** Fatima Zahra Jawhari, Abdelfattah El Moussaoui, Mohammed Bourhia, Hamada Imtara, Hamza Mechchate, Imane Es-Safi, Riaz Ullah, Essam Ezzeldin, Gamal A. Mostafa, Andriy Grafov, Samir Ibenmoussa, Dalila Bousta, Amina Bari

**Affiliations:** 1 Laboratory of Biotechnology, Environment, Agri-Food, and Health (LBEAS), Faculty of Sciences, University Sidi Mohamed Ben Abdellah (USMBA) Fez, Fes 30050, Morocco; abdelfatah.elmoussaoui@usmba.ac.ma (A.E.M.); hamza.mechchate@usmba.ac.ma (H.M.); imane.es@hotmail.fr (I.E.-S.); boustadalila@gmail.com (D.B.); amina.bari@usmba.ac.ma (A.B.); 2 Laboratory of Chemistry-Biochemistry, Environment, Nutrition, and Health, Faculty of Medicine and Pharmacy, University of Casablanca, B.P 5696, Casablanca 20250, Morocco; ibenmoussa@yahoo.fr; 3 Faculty of Arts and Sciences, Arab American University Palestine, P.O. Box 240, Jenin, Palestine; hamada.tarayrah@gmail.com; 4 Department of Pharmacognosy, College of Pharmacy, King Saud University, Riyadh 11451, Saudi Arabia; rullah@ksu.edu.sa; 5 Department of Pharmaceutical Chemistry, College of Pharmacy King Saud University, Riyadh 11451, Saudi Arabia; esali@ksu.edu.sa (E.E.); gmostafa@ksu.edu.sa (G.A.M.); 6 Department of Chemistry, University of Helsinki, P.O. Box 55, FI-00014 Helsinki, Finland; andriy.grafov@helsinki.fi

**Keywords:** *Anacyclus pyrethrum* (L), GC-MS analysis, analgesic, anti-inflammatory, wound healing

## Abstract

Background: *Anacyclus pyrethrum* (*A. pyrethrum*) is a wild species belonging to the family Asteraceae, which is used in traditional medicines. Aim of the study: This work was undertaken to study the chemical composition, analgesic, anti-inflammatory, and wound healing properties of hydroalcoholic extracts of different parts (roots, seeds, leaves, and capitula) of *A. pyrethrum.* Material and Methods: The phytochemical analysis of the studied extracts was conducted by GC-MS. The analgesic activity was evaluated in mice using acetic acid and formaldehyde methods. The anti-inflammatory activity was tested using the inhibitory method of edema induced in rats. The healing activity of the hydroethanolic extracts was explored by excision and incision wound healing models in rats. Results: The phytochemical analysis of the studied plant extracts affirmed the presence of interesting compounds, including some newly detected elements, such as sarcosine, *N*-(trifluoroacetyl)-butyl ester, levulinic acid, malonic acid, palmitic acid, morphinan-6-One, 4,5.alpha.-epoxy-3-hydroxy-17-methyl, 2,4-undecadiene-8,10-diyne-*N*-tyramide, and isovaleric acid. The extracts of different parts (roots, seeds, leaves, and capitula) exhibited promising anti-inflammatory, analgesic, and wound healing effects, with percentages of inhibition up to 98%, 94%, and 100%, respectively. Conclusion: This study might contribute towards the well-being of society as it provides evidence on the potential analgesic, anti-inflammatory, and wound healing properties of *A. pyrethrum*.

## 1. Introduction

The traditional use of medicinal plants as natural remedies against various pathologies has received full attention from the scientific community. In the last few decades, herbal medicines have been extensively used to fight diseases worldwide due to their efficiency, low costs, and few side effects [1]. Plants contain an immense variety of bioactive molecules for therapeutic, agri-food, and cosmetic uses [2]. For many years, medicinal plants have been considered a promising source of essential raw material for the discovery of natural compounds that are used as subsequent drugs to fight ailments [3].

*Anacyclus pyrethrum* (L) is commonly known as African pyrethrum, akarkarha, tigendesste, and igendess. It is a species belonging to the family Asteraceae, which is indigenous to Morocco, Algeria, and Spain [4,5]. This species includes the two varieties *Anacyclus pyrethrum* var. *pyrethrum* (L) and *Anacyclus pyrethrum* var*. depressus* (Ball) Maire [6,7]. 

In traditional medicine, the roots of *A. pyrethrum* (L) are recommended for treating toothache, salivary secretion, angina, digestive problems, lethargy, female infertility, and even paralysis of the tongue and limbs. They are used in the form of cream-based animal fats to treat gout and sciatica and keep illness away [8]. Other pharmacological and biological properties of *Anacyclus pyrethrum* (L) roots have been reported in the literature, such as sialagogue [9], aphrodisiac [10,11,12], immunostimulant [13,14], antidepressant [15,16], antimicrobial [17,18], insecticide [19], anesthetic [20], anti-inflammatory [21,22], anticonvulsant [10,23,24], antioxidant [25], antidiabetic [26,27], and memory enhancer [28] properties. 

This work refers to a study that aimed to investigate the chemical profile, analgesic, anti-inflammatory, and healing properties of different parts (roots, seeds, leaves, and capitula) of *Anacyclus pyrethrum* (L) endemic to Moroccan soil. Even though Manouze et al. (2017) studied the anti-inflammatory effect of *Anacyclus pyrethrum,* their work was limited to roots only, unlike in the present study, where we made a comparison between areal and root parts and even included samples from a different geographic area. 

## 2. Results

### 2.1. Analgesic Activity of Samples Demonstrated by the Acetic Acid Method

The extracts of different parts of *A. pyrethrum* (L) showed a significant analgesic effect against pain caused by acetic acid compared to the control lot, with an inhibition percentage that ranged from 12% to 94% (*p* < 0.001). Detailed results are presented in Table 1. 

The reference product diclofenac showed a pain inhibition percentage of 43%. Based on the analgesic activity evaluated by the acetic acid test (Table 1), we found that the fraction of *A. pyrethrum* roots (APPR) at a dose of 300 mg/kg was the most active, with an inhibition percentage of 94.10 ± 4.35%. The fraction of *A. pyrethrum* leaves (APPF) at a dose of 500 mg/kg was the least active when compared to the other samples, with an inhibition percentage of 12.00 ± 5.27%. Concerning the effect of the seed fraction (APPG), the effect increased in a dose-dependent manner, unlike in the root fraction (APPR), where we noticed a decrease in the effects when increasing concentrations. However, the leaves (APPF) and capitula (APPC) fractions used at 300 and 500 mg/kg, respectively, had the best effects compared to other doses, reducing the number of contortions. The contortions observed for these doses were significantly lower than those observed for the control group (*p* < 0.001).

### 2.2. Analgesic Activity of Samples Identified by the Formaldehyde Method

After the formaldehyde injection, the intensity of pain was recorded during the first phase (0–5 min) and the second phase (15–30 min) (Table 2).

The extracts of different parts inhibited the pain induced by formaldehyde in the paws of mice. In the first phase (0–5 min), the inhibition ranged from 67% to 94%. However, in the second phase (15–30 min), the inhibitory effect of extracts varied from 76 to 91% compared to the control group. Diclofenac and plant extracts showed similar effects of pain inhibition in the two phases.

### 2.3. Anti-Inflammatory Activity

Hydroalcoholic extracts from *A. pyrethrum* parts significantly prevented the evolution of the rat paw volume in all phases (1, 3, and 5 h) compared to the control group through two methods of treatment (*p* < 0.001) (Table 3). After the first hour, the inhibition percentage varied from 61% to 71% in the groups treated by oral gavage. While the groups were treated dermally, the inhibition percentage was more important and varied from 60% to 82%. At the fifth hour, the inhibition percentage significantly increased for all samples and reached 94% in the lots treated with APPC and APPG by oral administration, and 96% and 98% in the same lots dermally treated. The reference product of diclofenac significantly and progressively reduced edema by 94% with dermal application and 79% with oral administration.

### 2.4. Wound Healing Activity

The comparison of the mean wound area of groups treated with extracts and those treated with Madecassol (control group) showed a significant difference, which started becoming very clear after three days of treatment (*p* < 0.001) (Table 4). As shown in Table 4, which represents the wound healing activity of samples as a function of time, the wounds treated with a pomade prepared from APPC extracts (10% and 5%) healed after twelve and fourteen days of treatment, respectively. The healing for APPR (5%) and APPG (10%) started becoming very important after fourteen days of treatment. On the last day (20 days), complete healing was recorded for all extracts, and a reappearance of hair was noticed in the scars.

### 2.5. Phytochemical Identification of Plant Extracts

**Figure 1:**
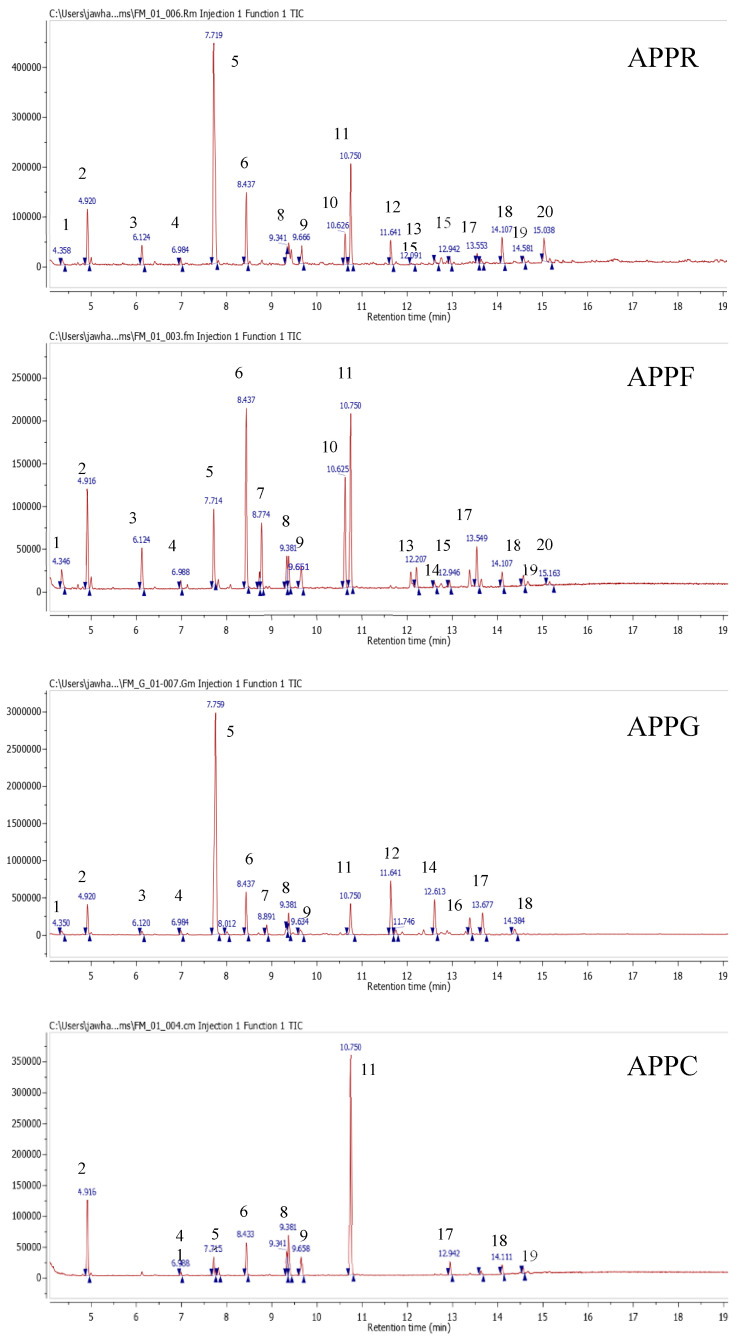
Chromatographic profile of different extracts (roots, seeds, leaves, and capitula) of *A. pyrethrum* (L).

The extracts of different parts (roots, seeds, leaves, and capitula) were analysed by GC/MS. As shown in Table 5 and Figure 1, GC/MS analysis confirmed the presence of twenty compounds in extracts studied, including *N*-isobutyl-dodeca-2,4,8,10-tetraenamide, *N*-isobutyl-2,4-octadiene-6-monoynamide, levulinic acid, propanedioic acid, palmitic acid, morphinan-6-One,4,5.Alpha.-epoxy-3-hydroxy-17-methyl, 2,4-undecadiene-8,10-diyne-*N*-tyramide, and dodeca-2*E*,4*E*,n*E*-trienoic acid 4-hydroxyphenylethylamide.

## 3. Discussion

The development of therapeutic agent-based natural products has become indispensable for modern medicines, in order to fight or prevent diseases. Medicinal herbs have historically demonstrated their value as an exhaustible source of potentially bioactive compounds, and nowadays, still represent an important reservoir for the identification of novel drug candidates [29].

In the present study, the hydro-ethanoic extracts of different parts of *A. pyrethrum* (L) had inhibitory effects on abdominal cramps resulting from the injection of acetic acid and the pain induced by the injection of formaldehyde solution. Pain caused by the injection of acetic acid and formaldehyde is due to the release of endogenous mediators that activate the nociceptive neurons, bradykinin, serotonin, cyclooxygenases, and their metabolites (e.g., PGE2 and PGF2a) in the peritoneal fluid. In addition, these solutions might activate peritoneal receptors and stimulate nociceptive nerve terminals [15]. In the present work, the analgesic effects of samples could be explained by the fact that the extracts studied might reduce the liberation of different inflammatory mediators such as serotonin, prostaglandins (PGE2 and PGEα), bradykinin, and histamine [30]. The analgesic effect investigated in this study is in accordance with the findings reported in previous literature [9,15,21,27]. Moreover, Rimbau et al. (1999) explained that the presence of alkamides in *A. pyrethrum* (L) extracts is responsible for the analgesic effect, inhibiting cyclooxygenase (COX) and 5-lipoxygenase (LOX) enzymes [31].

The injection of carrageenan induces the release of several inflammatory mediators, as follows: Serotonin and histamine within the first hour, and between 1.5 and 3 h, and bradykinin beyond the third hour [30]. Concerning the results of anti-inflammatory activity obtained in this study, the extracts found to reduce edema were significantly induced by carrageenan compared to the untreated groups and, therefore, we may affirm that the samples tested have an antagonistic effect on the liberation of responsible mediators for the inflammatory process. The phytochemicals detected in plant extracts are responsible for their anti-inflammatory effect induced by carrageenan and which may act, individually or in synergy, at different levels of the multifactorial process of inflammation [32]. Moreover, flavonoids have a membrane-stabilizing effect by reducing vasodilatation, which ameliorates the strength and integrity of blood vessel walls, while alkaloids may act through the prevention of neurogenic inflammation [33]. These results agree with those reported in the literature [21,23]. 

Regarding the wound healing activity, the macroscopic examination of wounds revealed a better evolution, with a substantial diminution in the wound diameter of treated animals with different extracts compared to both positive and negative controls. The healing effects of plant extracts could be attributed to their antibacterial effects, which ensure the protection of damaged tissues and the wound microenvironment from bacteria. Furthermore, the healing effects might also be due to the anti-inflammatory activity of plant extracts that manage immune cell accumulation at the wound site [29,34].

Chromatographic analysis of the studied extracts affirmed the presence of several components in the capitula, leaves, and seeds of *A. pyrethrum* (L) that may be involved in analgesic, anti-inflammatory, and healing activities. In the present work, twenty compounds were detected by GC-MS, and among them, sarcosine *N*-(trifluoroacetyl)-butyl ester, levulinic acid, propanedioic acid, palmitic acid, morphinan-6-one, 4,5 alpha-epoxy-3-hydroxy-17-methyl, 2,4-undecadiene-8,10-diyne-*N*-tyramide, and isovaleric acid compounds were detected for the first time in this work, according to the best of our knowledge. Some of the compounds identified in the studied extracts of *A. pyrethrum* (L), such as alkylamides ((2,4)-*N*-isobutyl-2,4-undecadiene-8,10-diynamide, *N*-isobutyl-dodeca-2,4,8,10-tetraenamide, *N*-isobutyl-2,4-octadiene-6-monoynamide, *N*-isobutyl-2,4-heptadiene-6-monoynamide, and N-isobutyl-2,6,8-datrienamide), were previously confirmed to have activities such as immunomodulatory, antithrombotic, antimicrobial, antiviral, antioxidant, anti-inflammatory, analgesic, anticancer, antidiabetic, and antiprotozoal activities [35,36,37,38]. Moreover, levulinic acid was found to have anti-inflammatory, anticonvulsant, and antioxidant activities [39,40,41,42]. Propanedioic acid was found to be an antimycobacterial, antimicrobial, anticancer, anticonvulsant, antiparasitic, antiviral, anti-HIV, anti-diabetic, antihypertensive, anti-hyperlipidemic, and monoamine oxidase inhibitor agent [43]. Cinnamic acid is well-known for its antioxidant, antitumor, antimicrobial, and antimycobacterial properties [44,45,46]. Pellitorin is an insecticidal, antibacterial, anticancer, anticoagulant, and anti-inflammatory agent [47,48,49]. Morphinan-6-One and 4,5.Alpha.-Epoxy-3-Hydroxy-17-Methyl have analgesic activities [50,51]. Isovaleric acid has a therapeutic effect as an antidyslipidemic anticonvulsant [3,52,53]. The richness of the studied extract in different chemical compounds with numerous activities, as reported in the earlier literature, could justify the obtained results in terms of the analgesic, anti-inflammatory, and wound healing properties, as described in the present work. 

## 4. Materials and Methods

### 4.1. Plant Samples

*A. pyrethrum* was harvested in July 2018 from the Timahdite region of Morocco. The plant was identified by a botanist and given the voucher number A31/31-5-18/TM. It has been deposited at the herbarium of the Department of Biology, Laboratory of Biotechnology, Environment, Agri-Food, and Health (LBEAS), Faculty of Sciences Dhar el Mahraz, Sidi Mohammed Ben Abdallah Fes University, Morocco. Different parts (roots, seeds, leaves, and capitula) of *A. pyrethrum* were washed with distilled water, separated, and dried in a shady place in a well-ventilated room for one week, before being sprayed and stored in bags away from light.

### 4.2. Extract Preparation

A total of 100 g of powder of different parts (roots, seeds, leaves, and capitula) of *A. pyrethrum* was extracted by maceration with 1000 mL of 70% ethanol at room temperature for 48 h. The mixtures were filtered and evaporated at 40 °C, and the residue obtained was then kept at 4 °C until further use. The extraction yield for roots and seeds was 16% and 10%, respectively, and a value of 14% was noted for both leaves and capitula extracts.

### 4.3. Preparation and Administration of Test Samples

Regarding oral administration, the crude extracts obtained were dissolved in distilled water and then stirred using a magnetic stirrer for 3 min. Afterward, they were orally administered to mice by using the gavage technique. The volume used for administration was determined according to the following formula [54]:

$$V=\frac{D\;\times \;P}{C}$$

where *V* is the volume of extract selected to be administered (mL), *D* is the dose (mg/kg), *P* is the weight of the animal (kg), and *C* is the concentration of extract selected to be administered (mg/mL).

Regarding topical administration, the preparation of cream from 5% and 10% extracts was conducted by incorporating 0.5 and 1 g of the studied extracts in 9.5 and 9 g of neutral cream, respectively. The topical administration was carried out by using cream from plant extracts (5% and 10%) [55].

### 4.4. Animals

Male adult Wistar rats (weighing 221 ± 22 g) and male Swiss mice (weighing 33 ± 3 g) were used to assess the pharmacological activities of plant samples. Animals were typically housed in cages (five animals/cage) in a temperature-controlled room with a 12/12 h light/dark cycle and relative humidity of 55 ± 5%. The animals were given free access to food and water. The institutional ethical committee of care and use of the laboratory animals at the Faculty of Sciences Dhar El Mehraz, Sidi Mohamed Ben Abdallah Fez University, Morocco, reviewed and approved the present study under the ethical clearance number 04/2019/LBEAS. 

### 4.5. Analgesic Activity of Samples Demonstrated by the Acetic Acid Method

The analgesic effect of different extracts was evaluated according to the method described by Ouédraogo et al. [56]. The mice were randomly divided into 14 groups, with five mice in each; each extract (roots, seeds, leaves, and capitula) was orally administered to mice in three different doses of 300, 500, and 1000 mg/kg with the time. The negative control group was treated with physiological water and the positive control group was treated with 100 mg/kg of paracetamol. One hour after the administration of each extract, the pain was provoked by an intraperitoneal injection of acetic acid solution at 0.6% (10 mL/kg) in each mouse. Five minutes after the injection, the number of contortions was counted for 30 min in each mouse. The percentage of pain inhibition was calculated according to the following formula:

$$\mathrm{Inhibition}\;\%=\left(1-\frac{C_{T}}{C_{Tn}}\right)\times 100$$

where C_Tn_ means number of contortions in mice from the negative control lot and C_T_ means number of contortions in mice from the treated lot. 

### 4.6. Analgesic Activity of Samples Revealed by the Formaldehyde Method

In this work, the formaldehyde method was also used to evaluate the analgesic effect, as described in earlier work [57]. Briefly, one hour before the injection of 20 μL of formaldehyde solution (2.5%) under the plantar pad of the right hind leg, groups were orally treated with APPR (300 mg/kg), APPF (300 mg/kg), APPC (500 mg/kg), and APPG (500 mg/kg). Paracetamol (100 mg/kg) was used as a reference product. Immediately after the injection of formaldehyde solution, the licking time (in seconds) of the treated paw was determined in two phases. The first phase was from 0 to 5 min, and the second was from 15 to 30 min, with an intermediate period of 10 min. The pain inhibition percentage was determined according to the following formula:

$$\mathrm{Inhibition}\;\%=\left(\frac{\mathrm{Lc}-\mathrm{Lt}}{\mathrm{Lc}}\right)\times 100_{\;}$$

where Lc is the average amount of licking of the control per group and Lt is the average amount of licking of the test per group.

### 4.7. Anti-Inflammatory Activity

The anti-inflammatory activity was evaluated by cutaneous and oral methods, according to Winter et al. [58]. Rats were divided into six groups, with five in each group. The negative control group was treated with physiological water, and the positive control group was treated with 1% diclofenac. One hour before the induction of inflammation by carrageenan (1%; NaCl 0.9%) under the plantar fascia of the right hind leg of rats, the volume of the paw was measured. Afterward, the paw was measured within 1 h, 3 h, and 5 h following the carrageenan injection.

The inhibition percentage of the inflammation was determined by the following formula:

$$\mathrm{Inhibition}\;\%=\;\left[\frac{\left(\mathrm{PAPc}\;-\;\mathrm{PAPt}\right)}{\mathrm{PAPc}}\;\right]\times \;100$$

where PAPc is the percentage increase in paw weight of the control lot and PAPt is the percentage increase in paw weight of the treated lot.

### 4.8. Wound Healing Activity

The wound healing activity was evaluated according to the method described by Imtara et al. [59]. Wounds were created on the skin of the dorsal-omoplate region of each anesthetized rat. The animals were divided into six groups, with five in each group. All groups were treated daily with cream extracts at 5% and 10% by applying them over the wound previously cleaned with alcohol at 96 °C. The negative control group was treated with neutral cream (containing no anti-healing molecules), and the positive control group was treated with Madecassol cream. The edges of wounds were traced on polypropylene sheets, and photographs were taken every day up to 21 days. The wound healing was regularly observed on days 1, 3, 7, 12, 15, and 18. Each wound area was calculated using Adobe Illustrator CS5 NA image analysis software (version 5).

### 4.9. Identification of Phytochemical Compounds

The determination of phytochemical compounds was carried out according to the silylation method described by Kabran and al. [31]. In short, 50 g of each sample (treated with petroleum ether and 250 mL of 2 N (HCl) was heated under reflux for two hours. After cooling, the hydrolysate was treated with 3 × 250 mL of ethyl acetate. The organic fractions were grouped, dried on anhydrous MgSO4, and then concentrated under vacuum. Afterward, 200 μL of *N*-methyl-*N*-trimethylsilyl trifluoroacetamide (MSTFA) was added to 3 mg of the resulting fraction and then heated at 37 °C for 30 min. Next, 0.1 μL of the sample was injected for analysis using a gas chromatograph coupled to a mass spectrophotometer (Brand Agilent Technologies Model 5973 with an Agilent column 19091S-433 HP-5MS, 30 m long, 0.25 mm inside diameter, and 0.25 μm film thickness of the stationary phase, (Helsinki, Finland) in positive mode. Helium was used as a carrier gas, with a typical pressure range (psi) of 0.9 mL/sec. The oven temperature program was set to 60–300 °C for 10 min and then maintained at 300 °C for 20 min. The detector temperature was set to 250 °C and the injector temperature to 260 °C. Identification of the silylated compounds was conducted by comparing the retention times with those of the standards obtained from the database.

### 4.10. Statistical Analysis

The results were expressed as means ± SEM. The analysis was performed via GraphPad Prism Software 6. Statistical processing was carried out by analysis of variance (ANOVA), followed by the Tukey multiple comparison test. A significant difference was considered at *p* < 0.05.

## 5. Conclusions

Different parts of *Anacyclus pyrethrum* (L) have important therapeutic activities, in terms of analgesic, anti-inflammatory, and healing activity, as described in this work. The *A. pyrethrum* capitula fraction was the most active extract in terms of the studied activities. Based on the results obtained in the present work, we may confirm that *Anacyclus pyrethrum* (L) possesses interesting natural chemical compounds that may serve society as analgesic, anti-inflammatory, and wound-healing agents. 

## Figures and Tables

**Table 1:** Analgesic activity results of different parts of *Anacyclus pyrethrum* (L) on abdominal contractions induced in mice by acetic acid injection.

Samples	Doses, mg/kg	Number of Contortions	Cramp Inhibition, %
Roots (APPR)	300	11.6 ± 4.35 ***	94.10 ± 4.35
	500	17.2 ± 6.13 ***	91.25 ± 6.13
	1000	51.6 ± 14.79 ***	73.75 ± 14.79
Seeds (APPG)	300	41.6 ± 11.79 ***	78.84 ± 11.79
	500	40.4 ± 13.21 ***	79.45 ± 13.23
	1000	24.4 ± 7.95 ***	87.59 ± 7.95
Leaves (APPF)	300	116.4 ± 8.91 **	40.79 ± 8.91
	500	173 ± 5.27	12.00 ± 5.27
	1000	141 ± 11.29 *	28.28 ± 11.29
Capitula (APPC)	300	66 ± 6.50 ***	66.42 ± 6.50
	500	58.4 ± 4.27 ***	70.29 ± 4.27
	1000	104.6 ± 4.49 **	46.79 ± 4.49
Control	-	196.6 ± 10.70	-
Diclofenac	100	111.8 ± 22.47 **	43.13 ± 22.47

* Correlation is significant at *p* < 0.05. ** Correlation is significant at *p* < 0.01. *** Correlation is significant at *p* < 0.001. Values are expressed as the mean ± SEM.

**Table 2:** Analgesic activity results of different parts of *A. pyrethrum* (L) and the effect of diclofenac (100 mg/kg) on the pain (licking time) induced by the injection of formaldehyde solution.

Samples	First Phase (0–5 min)		Second Phase (15–30 min)	
	Duration of Nociceptive Response (sec)	% Inhibition	Duration of Nociceptive Response (sec)	% Inhibition
Roots (APPR) (300 mg/kg)	16.2 ± 1.28 ***	88.31 ± 1.28	13.8 ± 0.96 ^***^	88.87 ± 0.96
Seeds (APPG) (500 mg/kg)	22.6 ± 5.83 ***	83.69 ± 5.83	29.2 ± 6.09 ^***^	76.45 ± 6.09
Leaves (APPF) (300 mg/kg)	8.2 ± 2.26 ***	94.08 ± 2.26	15.2 ± 2.22 ^***^	87.74 ± 2.22
Capitula (APPC) (500 mg/kg)	44.4 ± 2.50 ***	67.96 ± 2.50	10.8 ± 2.51 ^***^	91.29 ± 2.51
Control (Nacl)	138.6 ± 13.4	-	124 ± 2.23	-
Diclofenac (100 mg/kg)	97.8 ± 8.85 **	29.44 ± 8.85	67.4 ± 2.42 ^**^	45.65 ± 2.42

** Correlation is significant at *p* < 0.01. *** Correlation is significant at *p* < 0.001. Values are expressed as the mean ± SEM.

**Table 3:** Anti-inflammatory activity of different parts of *A. pyrethrum* (L) and the effect of diclofenac by oral and dermal administration on carrageenan-induced paw edema in rats.

Samples	Oral Administration						Dermal Administration					
	Oedema Volume (ΔmL)			% Inhibition			Oedema Volume (ΔmL)			% Inhibition		
	1 h	3 h	5 h	1 h	3 h	5 h	1 h	3 h	5 h	1 h	3 h	5 h
Roots (APPR) (300 mg/kg)	0.32 ± 0.04 ***	0.2 ± 0.03 ***	0.06 ± 0.02 ***	61.90 ± 4.45	73.68 ± 4.16	91.18 ± 3.60	0.22 ± 0.06 ***	0.14 ± 0.05 ***	0.04 ± 0.02 ***	76.09 ± 6.34	84.78 ± 5.54	96 ± 2.45
Seeds (APPG) (500 mg/kg)	0.24 ± 0.07 ***	0.14 ± 0.06 ***	0.04 ± 0.02***	71.43 ± 8.91	81.58 ± 7.89	94.12 ± 3.60	0.16 ± 0.04 ***	0.1 ± 0.031 ***	0.039 ± 0.02 ***	82.61 ± 4.35	89.13 ± 3.44	96 ± 2.45
Leaves (APPF) (300 mg/kg)	0.24 ± 0.02 ***	0.12 ± 0.02 ***	0.06 ± 0,02 ***	71.43 ± 2.92	84.21 ± 2.63	91.18 ± 3.60	0.36 ± 0.05 ***	0.26 ± 0.02 **	0.2 ± 0.04 **	60.87 ± 5.54	71.74 ± 2.66	80 ± 4.47
Capitula (APPC) (500 mg/kg)	0.28 ± 0.06 ***	0.14 ± 0.05 ***	0.04 ± 0.02 ***	66.67 ± 6.94	81.58 ± 6.71	94.12 ± 3.60	0.28 ± 0.05 ***	0.14 ± 0.05 ***	0.02 ± 0.02 ***	69.57 ± 6.34	84.78 ± 5.54	98 ± 2
Control (Nacl)	0.9 ± 0.03	0.98 ± 0.04	0.94 ± 0.05	0	0	0	0.92 ± 0.02	0.92 ± 0.05	1 ± 0.04	0	0	0
Diclofenac (100 mg/kg) or cream 1%	0.64 ± 0.08 *	0.26 ± 0.04 **	0.14 ± 0.04 **	23.81 ± 8.91	65.79 ± 5.26	79.41 ± 5.88	0.52 ± 0.08 **	0.28 ± 0.07 **	0.04 ± 0.02 ***	38.10 ± 9.52	63.18 ± 9.67	94.12 ± 3.60

* Correlation is significant at *p* < 0.05. ** Correlation is significant at *p* < 0.01. *** Correlation is significant at *p* < 0.001. Values are expressed as the mean ± SEM.

**Table 4:** The results of the wound healing activity of different parts of *A. pyrethrum* (doses of 5% and 10%) and Madecasol compared to the negative control.

Samples	Doses	Day 1	Day 3	Day 7	Day 12	Day 14	Day 16	Day 18
Roots (APPR)	5%	0	17.52 ± 3.00	74.45 ± 2.08	99.81 ± 0.19	100 ± 0	100 ± 0	100 ± 0
	10%	0	10.73 ± 1.84	59.35 ± 4.94	98.23 ± 0.75	99.84 ± 0.16	100 ± 0	100 ± 0
Seeds (APPG)	5%	0	10.08 ± 1.05	55.93 ± 1.21	96.07 ± 0.26	99.5 ± 0.04	100 ± 0	100 ± 0
	10%	0	14.26 ± 0.46	60.71 ± 3.22	98.93 ± 0.11	100 ± 0	100 ± 0	100 ± 0
Leaves (APPF)	5%	0	10.15 ± 1.28	44.74 ± 1.87	86.19 ± 1.95	95.46 ± 0.52	99.04 ± 0.29	100 ± 0
	10%	0	11.69 ± 1.67	41.79 ± 4.05	79.16 ± 2.44	94.17 ± 2.21	99.58 ± 0.22	100 ± 0
Capitula (APPC)	5%	0	14.47 ± 1.78	56.29 ± 1.40	98.89 ± 0.422	100 ± 0	100 ± 0	100 ± 0
	10%	0	16.09 ± 3.01	73.54 ± 6.43	100 ± 0	100 ± 0	100 ± 0	100 ± 0
Control	-	0	5.90 ± 0.62	34.57 ± 2.93	66.14 ± 2.48	75.25 ± 2.50	84.88 ± 1.40	92.73 ± 1.66
Diclofenac 1%	-	0	12.54 ± 2.22	44.15 ± 4.31	77.47 ± 3.34	89.14 ± 1.58	96.22 ± 1.31	100 ± 0

Values are expressed as the mean ± SEM.

**Table 5:** Chemical composition of different parts (roots, seeds, leaves, and capitula) of *A. pyrethrum* (L).

N°	RT	*m*/*z* Quasi-Molecular Peak	Structural Formula	Compounds	% Area			
					*Anacyclus pyrethrum* (L)			
					Roots (APPR)	Seeds (APPG)	Leaves (APPF)	Capitula (APPC)
1	4.35	231 (M + H)^+^	C_15_H1_9_NO	(2,4)-*N*-isobutyl-2,4-undecadiene-8,10-diynamide	0.97	0.76	2.29	-
2	4.92	246 (M)^+^	C_16_H_25_ON	*N*-isobutyl-dodeca-2,4,8,10-tetraenamide	6.79	4.44	9.45	15.91
3	6.12	241 (M)^+^	C_9_H_14_F_3_NO_3_	Sarcosine, *N*-(trifluoroacetyl)-, butyl ester	2.65	0.65	4.26	-
4	6.98	193 (M + H)^+^	C_12_H1_7_ON	*N*-isobutyl-2,4-octadiene-6- monoynamide	0.76	0.68	0.68	0.74
5	7.71	116 (M)^+^	C_5_H_8_O_3_	Levulinic acid	37.47	50.45	7.01	3.66
6	8.43	104 (M)^+^	C_3_H_4_O_4_	propanedioic acid	8.48	6.39	16.86	6.50
7	8.77	177 (M)^+^	C_11_H_15_ON	*N*-isobutyl-2,4-heptadiene-6- monoynamide	-	1.52	6.29	-
8	9.38	256 (M)^+^	C_16_H_32_O_2_	Palmitic Acid	2.85	2.75	3.17	8.34
9	9.65	285 (M)^+^	C_17_H_19_NO_3_	Morphinan-6-One, 4,5.Alpha.-Epoxy-3-Hydroxy-17-Methyl	2.17	1.31	2.86	4.93
10	10.62	147 (M)^+^	C_9_H_8_O_2_	Cinnamic acid	-	-	10.53	-
11	10.75	278 (M + H)^+^	C_18_H_31_NO	2,4-undecadiene-8,10-diyne-*N*-tyramide	11.09	5.34	16.50	46.07
12	11.64	271 (M)^+^	C_18_H_25_NO	*N*-isobutyl-dodeca-2,4,8,10-tetraenamide (Anacycline)	2.94	8.63	-	-
13	12.12	221 (M)^+^	C_14_H_23_NO	*N*-isobutyl-2,6,8-decatrienamide	0.63	-	2.06	-
14	12.61	223 (M)^+^	C_14_H_25_NO	(2*E*,4*E*)-*N*-(2-methylpropyl)deca-2,4-dienamide (Pellitorine)	1.16	6.04	0.78	-
15	12.94	274 (M + H)^+^	C_18_H_27_NO	Tetradeca-2*E*-diny-8,10-diynoic acid IBA	0.77	-	0.59	2.72
16	13.39	270 (M + H)^+^	C_18_H_23_NO	Tetradeca-2*E*,4*E*, n*E*-trienoic-8,10-diynoic acid IBA	-	2.85	1.86	-
17	13.67	102 (M)^+^	C_5_H_10_O_2_	Isovaleric acid	1.28	4.13	4.14	-
18	14.10	313 (M)^+^	C_20_H_27_NO_2_	Dodeca-2*E*,4*E*, n*E*-trienoic acid 4-hydroxyphenylethylamide	3.54	1.15	1.58	2.08
19	14.57	251 (M)^+^	C_16_H_29_NO	2,8-*N*-isobutyl-2,8-dodecadienamide	0.87	-	1.19	1.19
20	15.15	341 (M)^+^	C_22_H_31_NO_2_	Tetradeca-2*E*,4*E*,8Etrienoic acid 4-hydroxyphenylethylamide	0.61	-	0.82	-

## Abbreviation

APPR	*Anacyclus pyrethrum* (L) roots
APPG	*Anacyclus pyrethrum* (L) seeds
APPF	*Anacyclus pyrethrum* (L) leaves
APPC	*Anacyclus pyrethrum* (L) capitula
